# Porous Silicon-Based Catalysts for the Dehydration of Glycerol to High Value-Added Products

**DOI:** 10.3390/ma11091569

**Published:** 2018-08-31

**Authors:** Juan Antonio Cecilia, Cristina García-Sancho, Carmen Pilar Jiménez-Gómez, Ramón Moreno-Tost, Pedro Maireles-Torres

**Affiliations:** Universidad de Málaga, Departamento de Química Inorgánica, Cristalografía y Mineralogía (Unidad Asociada al ICP-CSIC), Facultad de Ciencias, Campus de Teatinos s/n, 29071 Málaga, Spain; jacecilia@uma.es (J.A.C.); carmenpjg@uma.es (C.P.J.-G.); rmtost@uma.es (R.M.-T.); maireles@uma.es (P.M.-T.)

**Keywords:** mesoporous silica, glycerol, dehydration, acid catalysis, acrolein

## Abstract

Increasing worldwide biodiesel production has led to the generation of an important glycerol surplus, which needs to be valorized in order to improve the economic and environmental sustainability of the biodiesel industry. In this context, glycerol dehydration to acrolein by acid catalysis appears to be a potential route of glycerol valorization, since acrolein is an important intermediate for many chemical industries. The main drawback of this catalytic process is catalyst deactivation. Different alternatives have been proposed for overcoming it, such as the use of mesoporous materials in order to facilitate the diffusion of glycerol and reaction products, thus minimizing deactivation. This review compiles the main achievements of the use of mesoporous silica-containing materials that have been deployed either as a catalyst or for support in glycerol dehydration to acrolein. Thus, the effect of mesoporosity on both catalytic performance and deactivation will be discussed, as well as the blocking of pores by coke deposition.

## 1. Introduction

**Figure 1:**
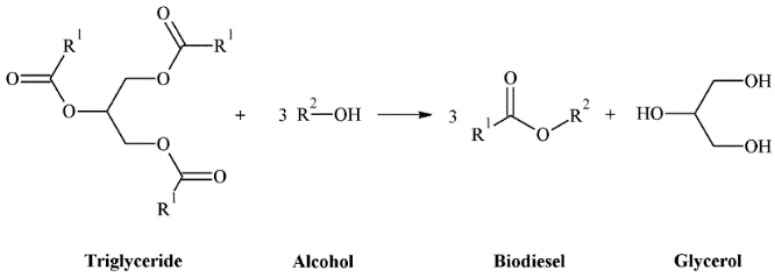
Transesterification reaction for biodiesel synthesis.

The growth of the world population, as well as improvement in quality of life, have caused an exponential consumption of fossil resources in the last century. This has led to their depletion, so it the search and development of alternative energy sources to replace them efficiently is necessary. Among them, biomass is the only sustainable source from which energy, fuels, and chemicals can be obtained [1]. Currently, the governments are legislating stringent regulations in which traditional fuels must be blended with biofuels in order to decrease emissions associated with fossil fuels. In this regard, the European Union aims to have 10% of the transport fuel coming from renewable sources in order to reduce greenhouse gas emissions [2]. Among them, the most important biofuels, in terms of production and mature industrial technologies, are bioethanol and biodiesel. In this latter case, it is produced from the transesterification process of vegetable oils or animal fats with short-chain alcohols, mainly methanol, under either base or acid catalysis, using homogeneous or heterogeneous conditions (Figure 1).

Biodiesel is an environmentally friendly fuel with a relatively low greenhouse effect, because of the lower pollutant gases and particulate matter emissions. In addition, biofuels avoid sulfur emissions and the formation of polycyclic aromatic hydrocarbons, and they are biodegradable in comparison to traditional fossil fuels [3,4].

The triglycerides used for the synthesis of biodiesel (Table 1) are traditionally obtained from several oleaginous plants, such as soybean, palm, sunflower or rapeseed. However, these vegetable oils are also used in food industries; therefore, they must not be considered as sustainable raw materials, since they could increase speculation, social imbalances, and the over-exploitation of fertile zones. Thus, the search for alternative oils is necessary, which should not interfere with the food chain. In this sense, an alternative feedstock for the synthesis of biodiesel is used as cooking oil, which is a waste without commercial interest.

**Figure 2:**
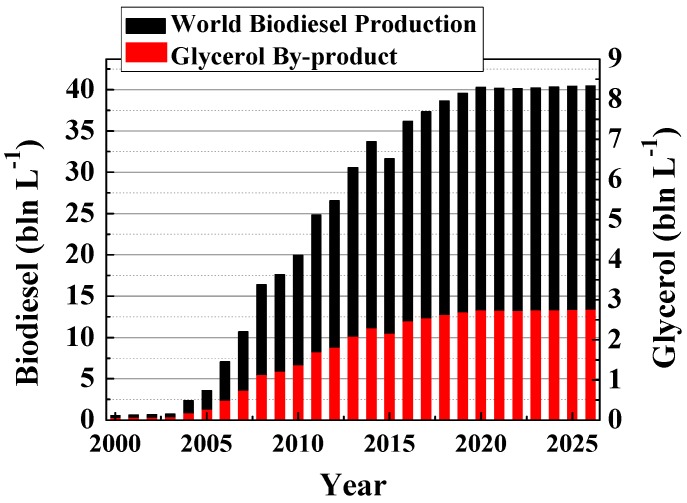
World production of biodiesel and glycerol (predictions of Organisation for Economic Co-Operation and Development/Food and Agriculture Organization of the United Nations (OECD-FAO)) [7].

The transesterification of triglycerides with short-chain alcohols generates biodiesel and glycerol as byproducts (Figure 1), in such a way that about a 10 wt.% of glycerol, with respect to biodiesel, is produced [6]. In recent years, the worldwide production of biodiesel is growing progressively (Figure 2) [7], and glycerol production has also risen consequently, so this in-stock glycerol should have a commercial outlet to improve the sustainability of biodiesel production.

Thus, glycerol can be transformed into value-added products. In this context, the catalytic dehydration of glycerol can yield acrolein, which is a versatile intermediate that is largely employed in the chemical industry. The presence of acid catalysts is needed to provide a suitable control of the reaction for attaining a significant acrolein yield. However, it has been demonstrated that the catalytic activity depends on the textural properties, mainly the pore size, which directly affects the deactivation processes [8,9,10].

The present review is included within a special issue titled “Mesoporous Silica Catalysts”, and this will be focused on the use of catalysts in which the mesoporous silica plays an important role, as support of the active phase or as catalyst itself, in glycerol valorization via its transformation to acrolein.

## 2. Glycerol Valorization

Glycerol is an organic molecule with three hydroxyl groups, which make glycerol very soluble in polar solvents, such as water and alcohols. Moreover, the hydroxyl groups form an extensive network of hydrogen bonds, as their boiling temperature is high in relation to their molecular size. Moreover, its presence provides glycerol molecules with a high versatility and physicochemical properties, allowing direct applications for facilitating the synthesis of high value-added chemicals.

Nowadays, glycerol finds more than 1500 applications across several industries, including food, pharmaceutical, personal care, antifreeze, and vibration dampening, among others [8]. In fact, the United States Department of Energy has considered glycerol to be one of the major building block chemicals [11]. Despite its high potential, the current situation is different, since the crude glycerol obtained from triglycerides transesterification has a low quality, which is one of the reasons for the marginal price of this byproduct. For this reason, it is generally burned, thus wasting this organic raw material with a high potential for obtaining a large spectrum of valuable chemicals.

**Figure 3:**
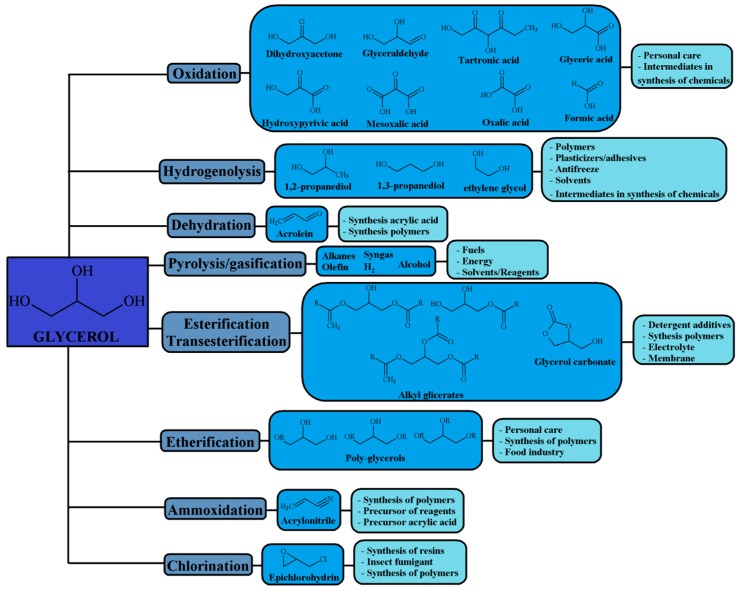
Valorization of glycerol and applications of some value-added products.

In this sense, the high glycerol reactivity allows its oxidation, partial or total hydrogenation, dehydration, and esterification or etherification, among other chemical transformations (Figure 3). In addition, glycerol can incorporate other heteroatoms, such as chorine by chlorination, or nitrogen by ammoxidation [12,13,14]. Among all this plethora of valorization routes, the present review will be focused on dehydration reactions. 

## 3. Glycerol Dehydration

One of the most interesting reactions for glycerol valorization is its dehydration to yield acrolein. This commodity is an important intermediate for the synthesis of methionine, methylpyridines, acrylic acid and esters, detergents, polymers, and super-adsorbents, and it can also be employed as biocide [8,14].

The glycerol dehydration to acrolein has been studied in both gas and liquid phases, although several authors have established that the gas phase is a more sustainable process, due to the operational and environmental issues of liquid phase reactions [15,16]. The glycerol dehydration requires acidic conditions, since the protonation of glycerol causes a decrease of the activation energy barrier from about 60 KJ mol^−1^ to 20 KJ mol^−1^ [17]. In this context, a relationship has been established between the strength of acid sites, according to the Hammett acidity, and the catalytic activity [18,19,20]. Thus, catalysts with the highest selectivity toward acrolein were those whose Hammett acidity was in the range −3 < Ho < −8.2. Several solid acid catalysts, such as metal sulfates [18,21,22] and phosphates [23,24,25], metal oxides (Al_2_O_3_, ZrO_2_, TiO_2_, WO_3_, Nb_2_O_5_, Ta_2_O_5_), zeolites [18,26,27,28,29,30,31,32,33,34,35,36], and heteropolyacids [18,37,38,39,40], have shown to be active in glycerol dehydration, with a high selectivity to acrolein. Among these catalysts, metal sulfates and phosphates or heteropolyacids exhibit mainly Brönsted acidity, while others such as Al_2_O_3_ or ZrO_2_ can be considered Lewis acid catalysts. Nevertheless, the most common is the coexistence of both Brönsted and Lewis acid sites, although it is not still clear which type of acidity is the most beneficial for glycerol dehydration.

**Figure 4:**
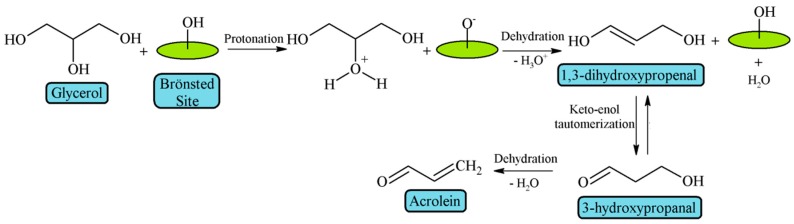
Mechanism of glycerol dehydration on Brönsted acid sites (adapted from Alhanash et al. [39]).

Alhanash et al. [39] established that Brönsted acid sites favor the protonation of the secondary hydroxyl group of glycerol, which is easily dehydrated to 1,3-dihydroxypropenal and then, after a keto-enol tautomerization, it is converted to 3-hydroxypropanal, whose subsequent dehydration leads to acrolein (Figure 4).

**Figure 5:**
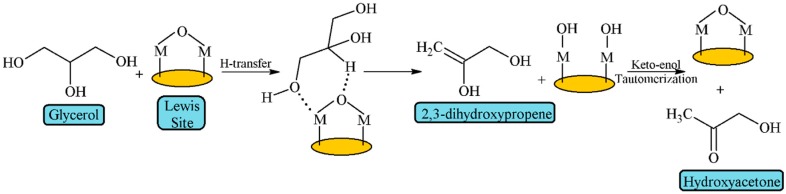
Mechanism of glycerol dehydration on Lewis acid sites (adapted from Alhanash et al. [39]).

On the other hand, Lewis acid sites preferentially activate the terminal hydroxyl groups due to the steric hindrance [39] (Figure 5). The interaction between a terminal OH group and a Lewis acid site favors the H transfer from the secondary C atom to the bridging O atom of the metal oxide, giving rise to 2,3-dihydroxypropene, which tautomerizes to acetol (hydroxyacetone), and the Lewis acid site is regenerated by thermal dehydration.

It can be observed that Lewis acid sites are transformed during the formation of 2,3-dihydroxypropene into pseudo-Brönsted acid sites, which can also lead to acrolein, or be regenerated to act as Lewis acid, favoring the formation of acetol [8].

**Figure 6:**
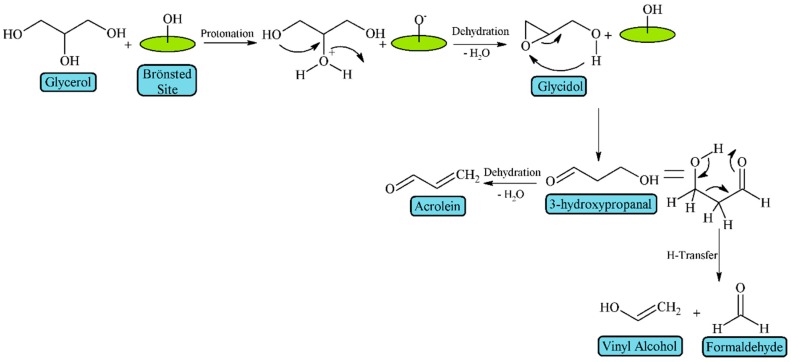
Alternative mechanism of glycerol dehydration on Brönsted acid sites (adapted from Laino et al. [41]).

On the other hand, Laino et al., from Density Functional Theory (DFT) studies, have proposed an alternative mechanism for glycerol dehydration to acrolein [41] (Figure 6). They suggest that the reaction takes place on Brönsted acid sites, generating an epoxide (glycidol) prior to the formation of 1,3-dihydroxypropenal. Then, this aldehyde is dehydrated to form acrolein, or an intramolecular hydrogen-transfer reaction can occur, leading to formaldehyde and vinyl alcohol.

However, it has been reported that other factors influence the catalytic performance besides the catalyst acidity, such as its textural properties. Thus, Tsukuda et al. [9] evaluated different supported heteropolyacids as catalysts for glycerol dehydration to acrolein, demonstrating that the catalytic activity depended on the type of heteropolyacid and on the size of mesopores of the silica support. They reported that silicotungstic acid supported on silica with mesopores of 10 nm exhibited a stable catalytic activity, obtaining a C_gly_ (glycerol conversion) = 100% and Y_acrol_ (Acrolein Yield) = 86%) in such a way that the size of the mesopores affected the catalytic activity, since small mesopores of 3 nm induced deactivation (C_gly_ = 55% and Y_acrol_ = 36%). Likewise, Pathak et al. [10] studied Zeolite Socony Mobil-5 (ZSM-5) zeolites with different pore sizes (0.54 nm, 0.74 nm, 3.15 nm, and 11.2 nm) and detected that the acrolein selectivity enhanced with the pore size, although selectivity toward other byproducts, such as formaldehyde, acetaldehyde, or hydroxyacetone, also increased. Therefore, the presence of mesopores seems to be directly related to the catalytic activity.

## 4. Mesoporous Silica-Based Catalysts Used in the Glycerol Dehydration

In this section, the most relevant silica-based catalytic systems studied for glycerol dehydration will be reviewed, in particular those employing a mesoporous silica as support or catalyst.

### 4.1. Mesoporous Silica

Since the Mobil company (Irving, TX, Estados Unidos) discovered the M41S family of mesoporous materials in 1992, much attention has been paid to mesoporous silica due to its use as catalyst in the oil refining and petrochemical industry or its use as adsorbent in separation processes. The synthesis of these mesoporous solids is based on the use of cationic surfactants as structure-directing agents (SDA) for the formation of the hybrid organo-inorganic precursor, which after subsequent calcination, or surfactant extraction, gives rise to a mesoporous framework. The formation of micellar structures helps build up the siliceous framework by polymerization of the silica precursors. This methodology allows the synthesis of mesoporous solids with a narrow pore size distribution, ranging between 2–6.5 nm [42]. Later, Stucky et al. at the University of Santa Barbara synthesized a new ordered mesoporous silica, using triblock copolymers as SDA, originating materials with pore diameters between 5–30 nm [43]. Their high specific surface area together with a narrow pore size distribution favored the dispersion of active phases and the diffusion of molecules. The use of these mesoporous materials in glycerol dehydration is compiled in Table 2.

**Figure 7:**
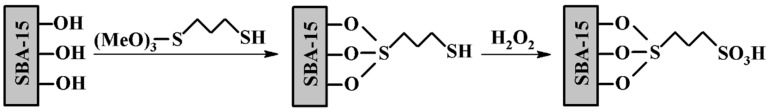
Functionalization of silica with sulfonic groups.

On the other hand, the presence of silanol groups (–Si–OH) on the silica walls allows the surface functionalization of the mesoporous silica by grafting. Thus, several authors functionalized the SBA-15 mesochannels by the grafting of mercaptopropyl trimethoxysilane. The –SH groups were then oxidized with H_2_O_2_ to generate sulfonic groups (SO_3_H) (Figure 7). These catalysts were active in the dehydration of glycerol due to the presence of Brönsted-type acidity on the surface of the mesoporous silica [44,45].

Lourenço et al. found that sulfonic acid groups incorporated to SBA-15 by the addition of 3-mercaptopropyltrimethoxysilane, which achieved a high acrolein yield (93%) and minimized the catalyst deactivation (Table 2). In addition, the large pore size of open channels of the SBA-15 silica favored the mass transfer and prevented deactivation [44]. These authors affirmed that both the pore size and density of acid sites played an important role in the deactivation rate and selectivity pattern, since a larger pore size significantly improved the catalytic activity, whereas a high density of acid sites could reduce acrolein production. However, Dalla Costa et al. observed a progressive deactivation under more severe reaction conditions (Table 2) [45]. They found that higher temperatures led to rapid deactivation due to a combined effect associated with coke deposition and the decomposition of sulfonic acid groups. Thus, temperatures above 300 °C provoked an irreversible deactivation. In both cases, the sulfonic acid groups were highly selective toward acrolein, with hydroxyacetone being a minor product and the density of sulfonic groups directly related to the catalytic activity.

Another strategy to generate Brönsted acid sites on the surface of mesoporous MCM-41 silica was by impregnation with phosphoric acid and subsequent calcination [46]. This catalyst followed the same trend as that observed for the mesoporous silica functionalized with sulfonic acid groups, since the main product was acrolein (Table 2) with a negligible amount of hydroxyacetone. This catalyst maintained the glycerol conversion (97%) during 10 h, but deactivation by coke deposition was detected for a longer reaction time, which was more pronounced than that observed for SBA-15 silica due to the narrower pore size of MCM-41, which favors a faster pore blockage. However, the H_3_PO_4_-modified MCM-41 catalyst recovered its activity after regeneration by a simple oxidative treatment in air.

Kobayashi et al. studied a mesoporous sulfated zirconia/silica catalyst with 20 wt.% SO_4_^2−^ in the catalyst, which was incorporated by using (NH_4_)_2_SO_4_ solutions [47]. They observed that sulfate groups provided Brönsted acid sites, although the most active and stable catalyst was that with a lower proportion of Brönsted acid sites, since its higher surface area minimized the formation of carbonaceous deposits. They proposed the existence of weak acid sites due to the dilution of zirconium species into silica, whereas the large pore size is suitable for a faster diffusion of glycerol, giving rise to better catalytic performance. The catalyst deactivation took place without the loss of sulfur, being ascribed to the covering of acid sites by coke.

The use of mesoporous silica also allows hosting and dispersing bulky polyoxocations, such as heteropolyacids, which enhance the acidity strength, especially of Brönsted acid sites. Thus, Ding et al. incorporated different loadings of H_3_PW_12_O_40_ into MCM-41 by impregnation, maintaining within the resulting solids a high specific surface area, determined by the Brunauer, Emmett and Teller (BET) equation, S_BET_ (415–624 m^2^ g^−1^) and pore size values (2.5–2.7 nm). The highest catalytic activity (C_gly_ = 85% and Y_acrol_ = 65%) (Table 2) was found for the catalyst with a 40 wt.% of H_3_PW_12_O_40_ [48]. Alike, Ma et al. compared two phosphotungstic acids (H_6_P_2_W_18_O_62_ and H_3_PW_12_O_40_) supported on MCM-41 by using the wet impregnation method [49]. They established from pyridine adsorption coupled to Fourier transform infrared (FT-IR) spectroscopy that a H_3_PW_12_O_40_/MCM-41 catalyst showed a higher total acidity, mainly of Lewis type, while H_6_P_2_W_18_O_62_/MCM-41 displayed the highest Brönsted:Lewis acid sites ratio. These data could be correlated with the catalytic behavior, since H_6_P_2_W_18_O_62_/MCM-41, with a higher proportion of Brönsted acid centers, was more selective toward acrolein (Table 2). On the other hand, the presence of a higher fraction of Lewis acid sites in H_3_PW_12_O_40_/MCM-41 catalyst caused a decrease in the acrolein selectivity, with a concomitant increase in hydroxyacetone. They also confirmed that catalyst deactivation was caused by both the leaching of HPW species and coke deposition.

One of the main drawbacks related to the use of heteropolyacids supported on mesoporous silica is related to their deactivation. Due to the high catalytic activity of this phase, leading to high acrolein yields, carbonaceous deposits are easily generated that block mesochannels, which gives rise to a faster deactivation associated with diffusional limitations. In this regard, several authors have proposed that the incorporation of Cs^+^ species into heteropolyacids supported on mesoporous silica can minimize the deactivation by coke deposition, since Cs^+^ partially neutralizes the strongest acid sites of heteropolyacids (Table 2) [50,51]. Other cations, such as Na^+^ or K^+^, were also proposed in order to reduce the formation of carbonaceous deposits; however, these cations drastically decrease the number of strong acid centers, which led to a lower catalytic activity and yield toward acrolein [51]. The deactivation also minimized incorporating O_2_ in the gas carrier, although the oxygen concentration had to be limited, since carbonaceous deposits could burn [50].

Other authors pointed out that the doping of heteropolyacids supported on mesoporous silica with noble metals, such as Pd or Pt, could reduce the coke, thus ameliorating the catalytic performance (Table 2) [52,53]. Trakarnpurk confirmed that the presence of platinum improved the long-term stability of a ZrMCM-41 impregnated with 2% Pt and 35% phosphotungstic acid catalyst [53]. The Pt-free catalyst exhibited an abrupt decrease in conversion (until 60% after 20 h of time-on-stream) due to coke deposition. In the same way, Ma et al. [52] demonstrated that a Pd-H_3_PW_12_O_40_/Zr-MCM-41 catalyst, prepared by impregnation, significantly enhanced the acrolein selectivity, with lower coke deposition and improved stability, with the support mesostructure remaining almost unchanged.

On the other hand, Viswanadham et al. established that the incorporation of vanadium species in the heteropolyacid structure also minimized the coke deposition, due to the redox properties of vanadium species retarding the deactivation [54]. These authors also observed a faster deactivation for the heteropolyacid supported in porous materials with a narrower pore diameter (Y-zeolite), while the use of large pore supports, such as SBA-15, maintained the catalytic activity for longer reaction times, because larger channels can avoid the pore blockage. However, the pore size distribution must be tuned to ensure an appropriate interaction between glycerol and the active centers.

The incorporation of heteroatoms, such as zirconium (IV) or aluminum (III), into a mesoporous silica is a classical methodological approach to generate acid sites for glycerol dehydration. Thus, the substitution of an Si (IV) atom by a heteroatom increases the acid strength of Si–OH groups, whereas the formation of M–OH–Si linkages, similarly to Si–OH–Al groups in zeolites, creates new Brönsted acid sites [55,62,63]. In this process, Lewis acid sites can be also formed, which are associated with deficiently coordinated heteroatoms. However, these Lewis acid centers can be transformed into pseudo-Brönsted sites by the water present in the reaction medium, as previously reported [8]. García-Sancho et al. synthesized a zirconium-doped mesoporous MCM-41 silica, incorporating Zr species into the siliceous framework, with different Si/Zr molar ratios (4–12), obtaining a maximum glycerol conversion of 97% with an acrolein yield of 39% after 2 h of time-on-stream at 325 °C for a Si/Zr molar ratio of five (Table 2) [55]. They also detected a high proportion of acetaldehyde, which can be formed by the retro-aldol fragmentation of 3-hydroxypropanal at reaction temperatures above 300 °C [52]. However, other authors established that acetaldehyde could result from the decomposition of hydroxyacetone [64]. Unfortunately, Zr-MCM-41 catalysts showed a rapid deactivation. Moreover, heteroatom-doped mesoporous silica has been used as a support of heteropolyacids. This work aims to weaken the strength of the Brönsted acid sites being associated with heteropolyacids by their interaction with the support. Consequently, the formation of carbonaceous deposits was reduced, leading to more stable catalysts [52,56]. It was also observed that the presence of zirconium into the siliceous framework favored the catalyst regeneration in comparison to the catalyst without zirconium [65].

Different metal oxides, such as V_2_O_5_ [57], Nb_2_O_5_ [58], and WO_3_ [59], have been incorporated into a mesoporous silica doped with zirconium by incipient wetness impregnation using vanadyl acetylacetonate, niobium oxalate, and ammonium metatungstate hydrate as precursor salts, respectively, causing an increase in both Lewis and Brönsted acidity, due to the interaction of zirconium species with metal oxides. Brönsted and Lewis acid sites, which are transformed into pseudo-Brönsted acid sites, favored acrolein formation and reduced their deactivation by coke. In all of the cases, the thermal decomposition of acrolein led to acetaldehyde as byproduct. It can be observed in Table 2 that supported Nb_2_O_5_ species were more selective to acrolein than V_2_O_5_ and WO_3_. Later, phosphorus species were incorporated to these metal oxides, obtaining an amorphous pseudo-heteropolyacid after calcination [57,59,60]. The incorporation of high phosphorus contents by using phosphoric acid solutions caused a pore blockage, so the catalytic processes in these cases could take place mainly on the external surface. In spite of the decay of the specific surface area, the density of acid sites increased, as well as the proportion of Brönsted acid sites, leading to a higher acrolein yield and a decrease in carbon deposits. Therefore, these catalysts were more stable along the time-on-stream, even if only active sites located on the external surface were involved in the catalytic process.

On the other hand, Cecilia et al. employed a low-cost SBA-15 synthesized from sodium silicate, and carried out a partial extraction of the silicon species from the siliceous framework using basic conditions [61]. Later, aluminum species were incorporated into the siliceous framework by mixing different volumes of a 1.2 M AlCl_3_∙6H_2_O aqueous solution with 25 mL of tetramethylammonium hydroxide aqueous solution. The existence of two kinds of aluminum sites was inferred from ^27^Al-NMR spectroscopy: tetrahedral Al located inside the silica walls and extra-framework octahedral Al species. Pyridine adsorption studies indicated that the tetrahedral aluminum generated Brönsted acid sites, while the presence of octahedral aluminum, whose amount rose with the Al loading, caused an increment of total acidity and the amount of Lewis acid sites, but the concentration of Brönsted acid sites decreased. Therefore, a higher proportion of aluminum did not produce an increase in tetrahedral aluminum, since firstly, the aluminum was introduced into the framework defects, and the excess remained on the external surface of SBA-15 as octahedral aluminum, which displayed a lower catalytic activity in glycerol dehydration to acrolein. Although the acrolein yield was lower than that obtained for other catalysts (Table 2) and the active sites suffered deactivation by coke deposition, this Al-SBA-15 catalyst could be interesting, since it is a simple and inexpensive catalyst and it could be reused, at least during three catalytic cycles after thermal regeneration.

### 4.2. Zeolites

Zeolites have demonstrated to be highly active in glycerol dehydration to acrolein, due to the existence of strong Brönsted acid centers in the microporous structure. The catalytic data reveal that the SiO_2_/Al_2_O_3_ ratio is not the only factor influencing glycerol conversion and the selectivity pattern, but there must be other factors involved in the catalytic performance, such as the type of zeolitic framework or the pore dimensions and topology [32,34,66]. In general, the catalytic activity was directly related to the amount and strength of Brönsted acid sites, although the dimensions of cavities played a key role in determining the resistance to deactivation [34,67,68,69]. As the current review is only focused on mesoporous silica catalysts, microporous zeolites used in glycerol dehydration will not be dealt with.

Kim et al. [34] compared the catalytic performance of H-β, H-ferrierite, H-ZSM-5, H-Y, and H-mordenite with different SiO_2_/Al_2_O_3_ molar ratios (SiO_2_/Al_2_O_3_ = 5.1–350). The glycerol conversion was strongly dependent on their external surface area. However, most of the pores, mainly micropores, were filled with carbonaceous species at the initial stage of this reaction, provoking the catalyst deactivation. Carriço et al. employed a MCM-22 with different SiO_2_/Al_2_O_3_ molar ratios (30–80) prepared by using silica and aluminum nitrate as solid acid catalyst [66], and concluded that its crystallinity, as well as its textural properties, worsen for higher SiO_2_/Al_2_O_3_ molar ratios. Although MCM-22 catalysts were essentially microporous, the presence of secondary mesopores was detected. However, they found that an increase in the SiO_2_/Al_2_O_3_ molar ratio was accompanied by a reduction of the external surface area and a decrease of the mesoporous volume. The catalytic data showed that the catalyst with the highest acidity was that with the lowest SiO_2_/Al_2_O_3_ molar ratio, reaching the best acrolein yield.

**Figure 8:**
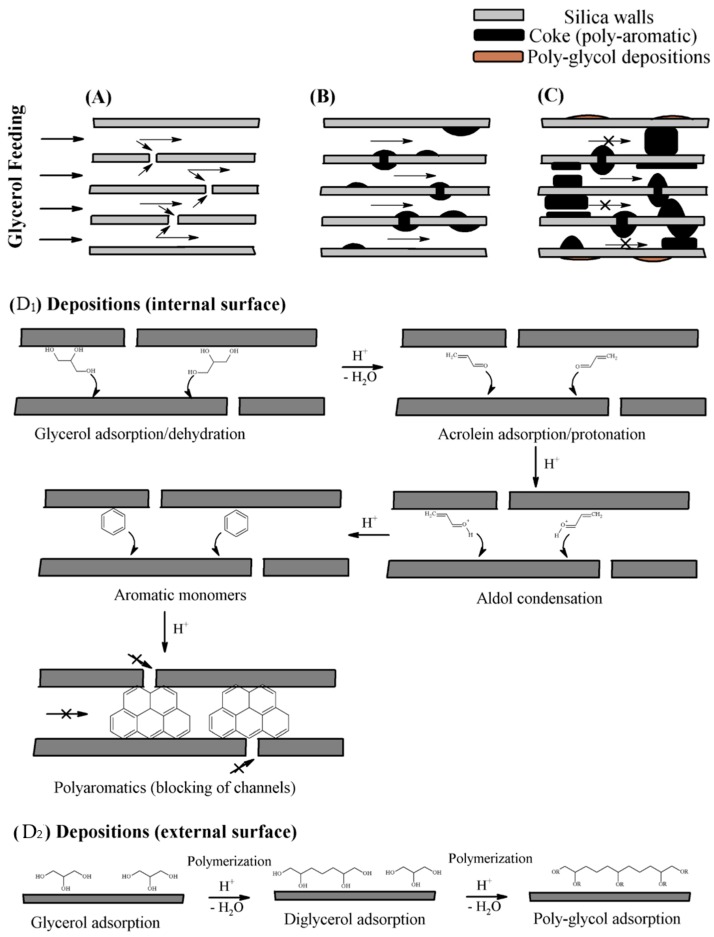
Scheme of the formation of carbonaceous deposits (**A**–**C**). Deposits formed on the internal surface (**D_1_**) and deposits formed on the external surface (**D_2_**).

Therefore, the coke deposition is directly related to the porous structure. Thus, zeolites with narrower pores are more susceptible to suffer pore blockage, and consequently they are deactivated more quickly. However, zeolites with larger pore volume, or open channels, are more resistant to blockage after short reaction times, since the diffusion is favored. Rodrigues et al. confirmed, by analyzing the carbonaceous deposits by ^13^C-NMR, the existence of aromatic and polyglycol deposits (Figure 8A–C) [68,70]. They concluded that polyaromatic species are formed inside pores on highly active Brönsted acid centers (Figure 8D_1_), in the first hours of reaction, whereas polyglycol deposits took place on the external surface of zeolites, since glycerol molecules were not able to access to the zeolitic cavities (Figure 8D_2_). 

In order to obtain more stable catalysts along the time-on-stream, the generation of mesoporosity by partial structural desilication, under basic conditions, has been proposed. Under these alkaline conditions, by using NaOH solutions, the SiO_2_/Al_2_O_3_ molar ratio should decrease, leading to a higher amount of available acid sites [71]. However, the desilication also caused the extraction of aluminum, which generated Lewis acid sites, so the selectivity to hydroxyacetone may increase. The catalyst stability was improved upon desilication due to an increase in coke tolerance. In addition, NH_3_-thermoprogrammed desorption (NH_3_-TPD) studies revealed a decrease of surface acidity strength, so the desilication had an antagonist effect [72]. Nonetheless, other authors pointed out that the Lewis acidity has a cooperative role with neighboring Brönsted centers, improving the catalytic activity [73]. In order to remove the extra-framework alumina associated with Lewis acidity, Lari et al. carried out a subsequent acid treatment to dissolve the aluminum debris, producing an improvement in the selectivity toward acrolein [74].

The coke deactivation can also be slowed down by synthesizing hierarchical zeolites with lower channel lengths. Thus, Zhang et al. evaluated the influence of the mesoporosity and acidity of hierarchical H-ZSM-5 zeolites on the catalytic performance, obtaining a higher coke resistance for the H-ZSM-5 with abundant open inter-crystalline mesopores, maintaining a full glycerol conversion after 14 h of time-on-stream (TOS) [75]. On the contrary, H-ZSM-5 catalysts with closed intercrystalline pores start to deactivate after 6 h of TOS [75]. Therefore, these results confirmed that an open and interconnected mesoporous architecture leads to a high activity, long lifetime, and improved selectivity, while the worse behavior of closed and small mesopores was attributed to the mass transfer limitations and/or the in-pore condensation of reactant or heavier reaction products. They also emphasized the need to design zeolites with proper hierarchical structure and acidity for maximal catalytic performance.

In the same way, Beerthuis et al. compared a commercial H-ZSM-5 with a hierarchical H-ZSM-5, micro-H-ZSM-5, and nano-H-ZSM-5 [76], being the catalytic results directly related to textural parameters. In this sense, the hierarchical H-ZSM-5 zeolite, with a higher pore volume and lower microporosity, reached the highest glycerol conversion and selectivity to acrolein, while the micro-H-ZSM-5, formed by microsized crystals with small micropores, suffered a faster deactivation by pore blocking. They related the improved stability and coking resistance to the hierarchical porosity formed by micropores, mesopores, and macropores. A similar result was reported by Huang et al., which synthesized a hierarchical H-ZSM-5 by an ultrasound-assisted method, obtaining a catalyst with longer life than the commercial catalyst [77].

Dos Santos et al. prepared ferrierite zeolites in fluoride medium as a mineralizing agent, which resulted in the formation of large crystals [78]. These authors compared the textural properties and catalytic behavior with that of a ferrierite synthesized by the classical method using a hydroxide medium. They found that the ferrierite zeolite synthesized in fluoride medium displayed a lower density of acid sites, which was probably due to the existence of Al–F bonds instead of Al–OH bonds; however, this catalyst was more active and stable than the traditional ferrierite.

Viswanadham et al. generated a high amount of Brönsted acid sites into a Y-zeolite by incorporating 10–40 wt.% of H_3_PW_12_O_40_ using a wetness impregnation method [79]. They attained the highest acrolein yield (79%) after 3 h of TOS at 275 °C for the catalyst with a 20 wt.% of H_3_PW_12_O_40_. However, the use of a higher loading of heteropolyacids did not improve the activity and/or the stability of the catalyst, which was explained by the partial collapse of the zeolite structure, as deduced from textural characterization. Thus, the acrolein selectivity decreased for higher phosphotungstic acid loadings due to the diffusional limitations associated with the smaller pore size.

Again, the incorporation of a low amount of noble metals helped to minimize the coke formation, delaying the catalyst deactivation. These noble metals can be catalytically active, giving rise to other high value-added products. In this sense, Lari et al. synthesized bifunctional catalysts based on noble metals (Ag, Au, Pd, Pt, and Ru) with a 5 wt.% metal loading supported on a Mordenite Framework Inverted (MFI) type zeolite treated under basic conditions with NaOH aqueous solutions to increase the dimensions of cavities [74]. They pointed out that the alkaline treatment generates an auxiliary network of intracrystalline mesopores, thus increasing the coking resistance and providing a longer lifetime in glycerol dehydration. The catalytic results revealed that the zeolite catalyzed the dehydration of glycerol to acrolein, while the noble metal acted as hydrogenation sites for acrolein, obtaining the highest allyl alcohol selectivity for the Ag-based catalyst and the highest propanal selectivity for the Pd-based and Pt-based catalysts.

**Figure 9:**
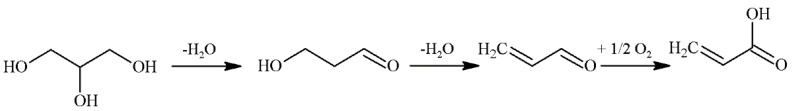
Reaction of glycerol oxidehydration to acrylic acid.

The incorporation of vanadium species in zeolites also exerts a beneficial effect on dehydration reactions due to the redox properties associated with vanadium, slowing the deactivation by coke. Moreover, the use of O_2_ as co-feed retarded the deactivation process and also favored the oxidation reaction of acrolein to acrylic acid in a process called oxidehydration (Figure 9). Acrylic acid is a valuable compound that is used in the manufacture of plastics, coatings, adhesives, and elastomers, as well as floor polishes and paints.

In a detailed study, several zeolites with different textural properties were impregnated with vanadium (5 wt.%) by using NH_4_VO_3_ solutions [80]. The use of N_2_/O_2_ as the carrier gas led to a negligible deactivation, except for those catalysts with a higher microporosity, or a higher SiO_2_/Al_2_O_3_ molar ratio, which suffered a slight deactivation after 10 h of TOS. In all of the cases, strong Brönsted acid sites associated with the zeolite framework were responsible for glycerol conversion to acrolein, whereas vanadium species oxidized by a Mars–van Krevelen cycle using mobile lattice oxygen, where V^5+^ was partially reduced to V^4+^ [70]. With regard to the selectivity, in all of the cases, acrolein was the main product, reaching yields between 12.7–46.8%, while acrylic acid was a minority product, obtaining a yield of 17.7% for V-β zeolite in the best case. These data followed the same trend as that shown for Pestana et al. for the same catalyst under milder reaction conditions [81], and also were in agreement with data obtained for V/MFI (SiO_2_/Al_2_O_3_ = 40) catalysts [70].

### 4.3. Silicon-Aluminophosphate (SAPO)

Aluminophosphates (AlPO_4_-n, where n denotes a structure type) were described by Wilson et al. in 1982 as a new class of zeolite-like molecular sieves [82]. These microporous materials are built up from a trick alternation of AlO_4_ and PO_4_ tetrahedra through corner sharing to form a neutral open framework. The neutrality of the AlPO_4_ structure limits its catalytic applications in dehydration reactions due to the absence of Brönsted acid sites. However, the substitution of phosphorus by silicon in the framework modifies the physicochemical properties, generating Brönsted acid sites [83], which are necessary for glycerol dehydration.

Several SAPOs (SAPO-11, SAPO-34, and SAPO-40) were evaluated in glycerol dehydration [84]. The catalytic results revealed a significant deactivation along the time-of-stream, being more pronounced in the case of SAPO-11 and SAPO-34 (Table 3). Lourenço et al. also indicated that the deactivation was directly related to the porous structure. The deactivation of SAPO-34 was more abrupt than that of SAPO-40 in spite of their similar microporous volume, due to the cage openings of SAPO-34 being much smaller than the pores of SAPO-40, leading to pore blocking by coke formation. In any case, the acrolein yield was higher than that previously reported by Chai et al. for SAPO-34 at short reaction times [18]. In the case of SAPO-11, an intermediate behavior was observed, since pore architecture influenced both the catalytic performance and the deactivation. Therefore, it seems clear that the porous network of solid acid catalysts had a strong influence on glycerol dehydration. Thus, for instance, SAPO-40, with a more open structure and a higher S_BET_ value, proved to be highly resistant under the experimental conditions used, and it was regenerated without a loss of activity or significant structural damages.

Regarding the selectivity, SAPOs showed similar acrolein selectivity, about 75%, and the presence of hydroxyacetone and acetaldehyde was also noteworthy (about 10% of selectivity) [84]. These data are in disagreement with those reported by Suprun et al., where the hydroxyacetone selectivity was higher [25]. They reported that SAPO-11 and SAPO-34 catalysts, with small micropores (5–6 Å), were less active but more selective than mesoporous Al_2_O_3_–PO_4_ and TiO_2_–PO4. Moreover, a detailed study with SAPO-40 revealed that the use of high reaction temperatures (T > 350 °C) causes a drastic deactivation by the formation of carbonaceous deposits, although the catalyst can be easily regenerated, maintaining its catalytic performance [84,85].

Recently, Fernandes et al. synthesized a mesoporous SAPO-40, using an organosilane as an additional porogen agent, replacing part of the silica source (Cab-OSil-M5) in the synthesis gel by [3-(trimethoxysilyl)propyl]-octadecyldimethyl-ammonium chloride, increasing the external surface area and mesopore volume [85]. In comparison to traditional SAPO-40, the long-term stability of mesoporous SAPO-40 is notably higher, demonstrating the beneficial effect of mesoporosity. Thus, an increase in the pore volume diminishes the diffusional limitations caused by the blockage of the structure with carbonaceous deposits and facilitates the access to acid sites.

### 4.4. Commercial Silica

Generally, commercial silica displays a spherical morphology with high porosity, with a high specific surface area associated with interparticle voids. In spite of this porosity, the weak acidity of the silica is not enough to favor the glycerol dehydration to acrolein. Thus, SiO_2_ is usually employed as support or mixed with active oxides to disperse them instead of the active catalyst itself.

The morphology of the silica can be easily modulated in comparison to other oxides, which is an important parameter for attaining a suitable catalytic performance. Thus, Tsukuda et al. used a silica composed of spheres of 6 nm as support for several Keggin-type heteropolyacids (HPAs) (Table 4) [9]. These authors reported that all of the catalysts, except the catalyst with boric acid (H_3_BO_3_) provided high values of glycerol conversion, although the acrolein yield differed between them. Generally, the main product was acrolein, although acetaldehyde and hydroxyacetone were also detected. Moreover, it is noteworthy that the silica impregnated with phosphomolybdic acid (Q6-PMo-30) reached lower acrolein yield values because of molybdenum triggers oxidation/reduction reactions, causing the formation of other products such as acetaldehyde. In all of the cases, the activity of the HPAs was higher in comparison to SiO_2_/Al_2_O_3_ and H_3_PO_4_/SiO_2_ due to the presence of stronger Brönsted acid sites associated with HPAs [86]. The highest catalytic activity in glycerol dehydration was found for the silica impregnated with silicotungstic acid, and it was ascribed to both the higher thermal stability (500 °C) of this HPA and its strongest acidity in comparison to other HPAs [87].

In addition, Tsukuda chose this most active phase, the silicotungstic acid, to be supported in three silicas with different pore diameter (3 nm, 6 nm, and 10 nm) [9]. The catalytic results revealed that the silica with a lower pore diameter was more susceptible to undergo deactivation at shorter reaction times in comparison to the silica with a pore diameter of 10 nm. Therefore, they confirmed that the catalytic activity depended on both the nature of the heteropolyacid and the size of the mesopores of the silica support.

Considering the high activity of the silicotungstic acid (H_4_SiW_12_O_40_), Kim et al. supported it on SiO_2_-Al_2_O_3_, but with a low percentage of Al (Si_0.9_Al_0.1_O_x_) [88]. The highest acrolein yield was 54% after 2 h of TOS (Table 4). In this sense, it has been previously established that at low Al loadings, the aluminum can be incorporated into the silica framework under basic conditions [61], exhibiting physicochemical properties similar to those of zeolites, although lacking the microporous ordering. This fact was confirmed by NH_3_-TPD, which revealed that the acidity was directly related to the catalytic behavior. Therefore, the presence of HSiW in supported catalysts increased the acidity, thus providing higher catalytic activities than those of the corresponding supports. Both glycerol conversion and acrolein yield significantly decreased with increasing time-on-stream due to carbonaceous species being deposited on the strong surface acid sites, provoking a loss of accessible active sites and decreasing the BET surface area and pore diameter, which complicated the diffusion of products.

Kang et al. carried out an analogous study using phosphotungstic acid (H_3_PW_12_O_40_) supported on SiO_2_-Al_2_O_3_, obtaining similar results [40]. These authors pointed out that the incorporation of small amounts of Al increased the proportion of Brönsted acid sites, while the progressive addition of Al-species caused an increase of the Lewis acidity, which is not beneficial for the dehydration of glycerol to acrolein. In all of the cases, the mesoporous structure of supports was maintained after the incorporation of H_3_PW_12_O_40_.

Likewise, Kim et al. prepared SiO_2_-Al_2_O_3_ catalysts by the incorporation of Al^3+^ in basic medium [89], as was previously indicated for HPAs [40,88]. These authors also observed that a small amount of Al^3+^ could be incorporated into the silica structure generating Brönsted acid sites, which favored glycerol dehydration, whereas the Lewis acidity was associated with the extra-framework Al^3+^. Nonetheless, these values were below those obtained with HPAs under similar catalytic conditions [59], due to the HPAs displaying stronger Brönsted acid sites, which improved the catalytic performance in the dehydration of glycerol to acrolein.

In another study, Chai et al. also supported phosphotungstic acid on a commercial SiO_2_ and compared its catalytic behavior to HPW/ZrO_2_ catalysts [90]. Despite the textural and acidic properties of HPW/SiO_2_, the catalytic data showed that the acrolein yield was higher when ZrO_2_ was employed due to the stronger interaction between the ZrO_2_ and the Keggin anion (Table 4).

On the other hand, the silica can be mixed with other metal oxides with greater acid strength in order to increase the amount of available sites for glycerol dehydration. Massa et al. synthesized WO_3_-Nb_2_O_5_ supported on several oxides (SiO_2_, TiO_2_ and Al_2_O_3_). However, the catalytic data showed that those catalysts supported on SiO_2_ reached the lowest acrolein yields (47% for 0.5WO_3_-0.5Nb_2_O_5_/SiO_2_ catalyst) (Table 4), probably because SiO_2_ is the support with the weakest acidity [30]. In all of the cases, acrolein and hydroxyacetone were obtained by the coexistence of Brönsted and Lewis acid sites; however, the main product was acrolein due to the transformation of the Lewis acid sites into pseudo-Brönsted acid sites by the presence of H_2_O in the reaction medium [39].

Nb_2_O_5_ supported on a commercial SiO_2_ was also evaluated in this reaction, obtaining the best results for the catalyst with a 20 wt.% of Nb_2_O_5_, which also displayed the highest acidity [91]. In addition, it was noteworthy that the most active catalyst in the first hours was more susceptible to suffer deactivation due to the faster formation of carbonaceous deposits. Several research works have reported the coexistence of Lewis and Brönsted sites in the Nb_2_O_5_ [92]. As has been previously mentioned, the Lewis acid sites interact with primary alcohols, favoring the formation of hydroxyacetone, while the interaction of secondary alcohols with Brönsted acid sites leads to acrolein (Figure 5 and Figure 6) [39]. In addition, these authors established that Brönsted acid sites are also involved in intramolecular reactions, resulting in the formation of coke and consequently provoking the catalytic deactivation [92].

WO_3_/ZrO_2_ catalysts doped with silica were studied in glycerol dehydration. This tungstated zirconia contained both Brönsted and Lewis acid sites, so the main products are acrolein and hydroxyacetone [64]. It has been suggested that H^δ+^(WO_3_)_n_^δ−^ Brönsted acid sites were responsible for the selectivity toward acrolein [64,93]. Lauriol-Garbey et al. also reported that catalysts doped with silica showed to be more selective and stable catalysts, attaining full conversion and an acrolein yield of 65% after 8 h of TOS (Table 4) [64]. They affirmed that the deposition of SiO_2_ on ZrO_2_ favored the formation of larger mesopores and reduced the support basicity, minimizing the production of coke precursors, which was crucial to delay the deactivation of catalysts.

## 5. Conclusions

Different mesoporous materials have been evaluated as solid acid catalysts for glycerol dehydration to acrolein in order to overcome drawbacks associated with deactivation by coke formation. Porous silica-based catalysts, such as mesoporous silica, zeolites, silicon-aluminophosphates, and commercial silica have demonstrated that the presence of mesopores improved catalytic performance. In general, the existence of large pores increases the resistance to pore blocking, since the products diffusion is favored, decreasing the deactivation rate and providing a longer lifetime in the dehydration of glycerol. However, the deactivation of these materials finally took place by coke deposition, since the stability of these mesoporous silicon-based catalysts during their regeneration by thermal treatment was important. Therefore, both the pore size and density of acid sites played an important role in the catalytic performance and deactivation rates, as they were crucial to the control of these parameters to maximize acrolein production.

## Figures and Tables

**Table 1:** Current feedstocks for biodiesel production, based on Tan et al. [5].

Feedstock	Types	Remarks
First generation	- Palm oil - Rapeseed - Soybean oil - Sunflower oil - Peanut oil	- Food feedstock - Competition with the edible oil market - Impact on food markets and security - Large portions of land are required
Second generation	- Jatropha oil - Sea mango - Tobacco seed oil - Tallow - Frying oil	- Low competition with food - Environmentally friendly - Poor performance in cold temperature - Animal fats (biosafety problems) - Valorization of residue

**Table 2:** Catalytic activity of catalysts based on mesoporous silica in glycerol dehydration.

Active Phase	T (°C)	TOS (h)	C_gly_ (%)	Y_acrol_ (%)	Mmol_acrol_ h^−1^ g_cat_^−1^	Ref.
SBA-15-SO_3_H	300	3	100	93	10.5	[44]
SBA-15-SO_3_H	300	6	45	35	13.9	[45]
SBA-15-PO_3_H	320	2	97	81	3.71	[46]
Meso SiO_2_-ZrO_2_/SO_4_^2−^	250	2	99	81	10.9	[47]
H_3_PW_12_O_40_/MCM-41	320	2	85	68	3.72	[48]
H_3_PW_12_O_40_/MCM-41	320	5	87	44	3.59	[49]
H_6_P_2_W_18_O_62_/MCM-41	320	5	82	51	4.20	
Cs_2.5_H_0.5_PW_12_O_40_/MCM-41	300	2	100	86	8.47	[50]
Cs_2.5_H_0.5_PW_12_O_40_/MCM-41	300	30	100	88	24.08	[51]
MCM41-Zr	320	5	74	19	0.87	[52]
Pd-HPW/Zr-MCM-41	320	5	94	80	3.64	
Pt-HPW/Zr-MCM-41	320	5	83	62	2.82	
MCM41-Zr	300	5	66	18	5.31	[53]
Pt-HPW/Zr-MCM-41	350	5	82	70	20.31	
H_4_PMo_11_VO_40_/SBA-15	225	4	100	74	1.69	[54]
MCM41-Zr	315	5	97	38	5.28	[55]
MCM41-Zr	250	0–5	96	71	4.86	[56]
V_2_O_5_/MCM41-Zr	325	2	90	25	4.41	[57]
V_2_O_5_-P/MCM41-Zr	325	2	90	41	6.71	
Nb_2_O_5_/MCM-41-Zr	325	2	77	45	7.43	[58]
WO_3_/MCM-41-Zr	325	2	97	41	6.71	[59]
WO_3_-P/MCM-41-Zr	325	2	78	51	8.36	
Nb_2_O_5_-P/MCM-41-Zr	325	2	100	56	9.18	[60]
Al-SBA-15	325	2	94	35	5.74	[61]

TOS (Time On Stream). C_gly_ (Glycerol Conversion). Y_acrol_ (Acrolein Yield).

**Table 3:** Catalytic activity of SAPO-11, SAPO-34, and SAPO-40 in glycerol dehydration.

Catalyst	T (°C)	TOS (h)	C_gly_ (%)	Y_acrol_ (%)	Mmol_acrol_ h^−1^ g_cat_^−1^	Ref.
SAPO-11	350	2.5	100	73	2.4	[84]
		120	26	19	0.6	
SAPO-34	350	2.5	90	69	2.3	
		120	16	12	0.4	
SAPO-40	350	2.5	100	75	2.5	
		120	54	39	4.0	
SAPO-34	315	9	32	15	0.1	[18]
SAPO-11	280	1	88	55	0.9	[25]
SAPO-34	280	1	59	42	0.7	
Meso-SAPO-40	320	2.5	100	81	2.7	[85]
	320	120	87	68	2.3	

**Table 4:** Catalytic activity of commercial silica-based catalysts for the glycerol dehydration reaction.

Active Phase	T (°C)	TOS (h)	C_gly_ (%)	Y_acrol_ (%)	Mmol_acrol_ h^−1^ g_cat_^−1^	Ref.
SiO_2_-H_3_PO_4_	325	5	70	38	2.0	[9]
SiO_2_-H_3_BO_3_	325	5	15	0.3	0.02	
SiO_2_-PW	325	5	100	65	3.9	
SiO_2_-PMo	325	5	98	33	2.0	
SiO_2_-SiW	325	5	100	74	4.4	
SiO_2_-SiW	315	2	23	6	13.1	[88]
Si_0.9_Al_0.1_O_x_-SiW	315	2	97	54	127.3	
Al_2_O_3_-SiW	315	2	87	33	26.1	
SiO_2_-PW	275	10	16	4	78.3	[40]
Si_0.85_Al_0.15_O_x_-PW	275	10	43	20	83.6	
Al_2_O_3_-PW	275	10	13	3	13.0	
S_i0.8_Al_0.2_O_x_	315	2	43	21	50.7	[89]
SiO_2_-PW	315	10	20	11	3.3	[90]
ZrO_2_-PW	315	10	70	49	8.1	
0.5Nb_2_O_5_0.5WO_3_/SiO_2_	305	1	99	47	0.4	[30]
SiO_2_/WO_3_/ZrO_2_	300	8	100	65	0.5	[64]
